# New Records of Potent In-Vitro Antidiabetic Properties of *Dalbergia tonkinensis* Heartwood and the Bioactivity-Guided Isolation of Active Compounds

**DOI:** 10.3390/molecules23071589

**Published:** 2018-06-29

**Authors:** Van Bon Nguyen, San-Lang Wang, Ngu Truong Nhan, Thi Hanh Nguyen, Nguyen Phuong Dai Nguyen, Do Huu Nghi, Nguyen Manh Cuong

**Affiliations:** 1 Institute of Research and Development, Duy Tan University, Da Nang 550000, Vietnam; bondhtn@gmail.com; 2 Department of Science and Technology, Tay Nguyen University, Buon Ma Thuot City 630000, Vietnam; ngutruongnhan@gmail.com (N.T.N.); nguyenhanh2208.tn@gmail.com (T.H.N.); nguyendhtn@gmail.com (N.P.D.N.); 3 Department of Chemistry, Tamkang University, New Taipei City 25137, Taiwan; 4 Life Science Development Center, Tamkang University, New Taipei City 25137, Taiwan; 5 Graduate University of Science and Technology, Vietnam Academy of Science and Technology (VAST), Hanoi 122100, Vietnam; dohnghi@gmail.com; 6 Institute of Natural Products Chemistry, Vietnam Academy of Science and Technology (VAST), Hanoi 122100, Vietnam

**Keywords:** diabetes, *Dalbergia tonkinensis*, sativanone, formononetin, heartwood, inhibitors

## Abstract

Alpha-glucosidase inhibitory activity has been commonly used for the evaluation of antidiabetic property in vitro. The aim of this study is to investigate and characterize *Dalbergia tonkinensis* as a potential source of antidiabetic compounds. The screening of the active parts used, such as trunk bark, heartwood, and the leaves of *Dalbergia tonkinensis* indicated that all these extracted parts used with methanol demonstrated potent α-glucosidase inhibitory activity. The in vitro antidiabetic property of *Dalbergia tonkinensis* was notably recorded for the first time and showed activity (EC_50_ = 0.17–0.78 mg/mL) comparable to those of reported potent herbal extracts (EC_50_ = 0.25–4.0 mg/mL) and higher activity than that of acarbose, a commercial antidiabetic drug (EC_50_ = 1.21 mg/mL). The stability tests revealed that the heartwood of *Dalbergia tonkinensis* extract (HDT) possesses high pH stability with relative activity in the range of 80–98%. Further bioassay-guided purification led to the isolation of 2 active compounds identified as sativanone and formononetin from the ethyl acetate fraction and water fraction of HDT, respectively. These α-glucosidase inhibitors (aGIs) show promising inhibition against various types of α-glucosidases. Remarkably, these inhibitors were determined as new mammalian aGIs, showing good effect on rat α-glucosidase. The results suggest that *Dalbergia tonkinensis* is a potent source of aGIs and suggest promise in being developed as functional food with antidiabetic efficacy. The results of this study also enrich our knowledge concerning current biological activity and constituents of *Dalbergia tonkinensis* species.

## 1. Introduction

Diabetes mellitus (DM), a major global health problem, has been quickly increasing, and has reduced the life quality of people worldwide [1]. In 2013, the number of people living with DM was reported to be 382 million; of these, 90% were affected by Type 2 diabetes and the estimated number of DM cases will reach 592 million by 2035 [2]. People affected by DM were reported to be at high risk of various diseases, including kidney failure, cardiovascular disease, depression, frailty, premature death, cognitive decline, and other diseases [1]. This disease has also been recognized as a major cause of death, accounting for around 8.4% of total global deaths in 2013 [3]. Therefore, this issue is of great interest to researchers worldwide. Several therapies for type 2 diabetes have been investigated. Of these, the use of aGIs is considered to be an effective therapy for this global disease [4].

aGIs have been obtained from several sources, including chemical synthesis [5,6], herbs [7,8,9], or microbial conversion of fishery by-products [10,11,12,13], nutrient broth [14,15], and fermented soybeans [16,17,18]. Of these, herbal aGIs have received much attention for investigation due to their nature and safety in type 2 diabetes management [7,8].

Vietnam, a tropical country has been considered as the sixteenth most biodiverse country with more than 10,000 plant species. Of these, about 4000 species have been used as medicinal sources [19]. Therefore, there have been an increased interest in the screening and isolation of bioactive compounds from herbs in recent years [7,8,9,20,21,22]; however, very few new records of antidiabetic herbs and compounds have been reported from this biodiverse area [7,21]. Thus, the discovery of natural products with beneficial properties from Vietnam has been received with great interest. 

*Dalbergia* species are distributed in tropical and sub-tropical areas with more than 300 recognized species [23,24]. This genus has been reported to possess numerous novel bioactivities, including: anti-cancer, antimicrobial, anti-inflammatory, analgesic, anti-diarrheal, cardiovascular, spermicidal, and antipyretic activities [24]. In particular, *Dalbergia odorifera* species has been extensively investigated for its numerous chemical constituents and various reported biological activities, including α-glucosidase inhibition [25]. In contrast, *Dalbergia tonkinensis* species, a medium-size floral species with a height of 5–13 m and widely distributed in Vietnam [26], and found in Hainan Island of China [27] has been poorly studied regarding its biological activities and chemical constituents. Some previous studies have reported on the isolation and identification of various constituents from *Dalbergia tonkinensis* heartwood, as well as its antimicrobial activity [26,28,29]. However, based on recent reviews, there has been no report so far on the α-glucosidase inhibitory activity of this herb and its antidiabetic compounds.

In this study, *Dalbergia tonkinensis* was collected in the Dak Lak Province, Vietnam and its heartwood was found to possess potent α-glucosidase inhibitory activity. Thus, this part was used in conducting subsequent experiments on the isolation and identification of its active compounds. In the current study, the specific inhibitory activity of isolated inhibitors was also tested for characterization of their potential use in the management of type 2 diabetes.

## 2. Results and Discussion

### 2.1. New Records of Dalbergia tonkinensis Extracts as Potential Sources of aGIs

*Dalbergia* species has been considered as a valuable source of bioactive products, and the heartwood of these herbs has been used in traditional Chinese medicines for the treatment of several diseases, such as heart failure, myocardial fibrosis, myocardial infarction, myocarditis, and coronary failure [24,25,30]. To evaluate *Dalbergia tonkinensis* as a source of antidiabetic drugs, the heartwood of *Dalbergia tonkinensis* (HDT) was extracted with methanol; its inhibition against α-glucosidase was then tested. The activity was calculated and expressed as EC_50_ value, which was defined as the concentration of the sample (inhibitor) that inhibits 50% of enzymatic activity. Therefore, the lowest value that an inhibitor gets, the greatest inhibitory activity it possesses. As shown in Table 1, HDT demonstrated much stronger activity than that of acarbose, an antidiabetic drug with the EC_50_ values of 0.17 mg/mL and 1.21 mg/mL, respectively. Recently, some herbs collected in Dak Lak Province were reported as potential sources of aGIs [7,8,9,22]. Based on the summary presented in Table 1, HDT also showed promising activity (EC_50_ = 0.17 mg/mL) compared to other reported herbs (EC_50_ = 0.25–4 mg/mL).

**Figure 1:**
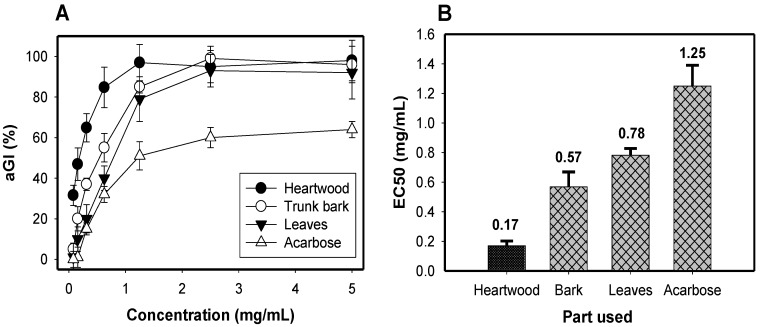
The inhibition of different *Dalbergia tonkinensis* parts used and expressed as aGI % (**A**) and EC_50_ values (**B**); results are means ± SD of multi tests (*n* = 3).

To determine which part used is the most active, the heartwood, trunk bark, and leaves of *Dalbergia tonkinensis* were collected and extracted with methanol; their activity was then tested. As shown in Figure 1A, all the parts used of *Dalbergia tonkinensis* show stronger activity (98–100%) than that of positive control (62%). These results highlighted the promising in vitro antidiabetic property of the extracts from the heartwood, trunk bark and leaves of *Dalbergia tonkinensis*. To clarify the results, the activity was also expressed as EC_50_ value. The heartwood extract demonstrated the strongest activity among the *Dalbergia tonkinensis* parts used due to its smallest EC_50_ value of 0.17 mg/mL; therefore, it was used in conducting subsequent experiments, including stability tests and purification.

### 2.2. The Stability of HDT against Different pH Treatments

**Figure 2:**
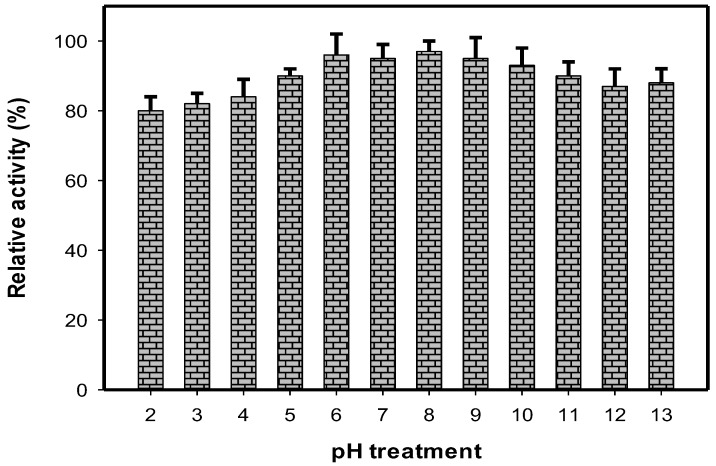
The pH stability of HDT. The sample was treated in the range of pH 2–13 at 37 °C for 30 min. The α-glucosidase inhibitory activity was then tested and expressed as relative activity (%). Yeast α-glucosidase was used for the enzymatic inhibition assay. Results are means ± SD of multi tests (*n* = 3).

It has been reported that a potential aGI should be stable under a wide range of pH readings, since the gastrointestinal tract is often acidic [7,10,14]. Thus, the pH stability of HDT was considered for testing in this study. As shown in Figure 2, the HDT showed its high pH stability with relative activity in the range of 80–98% after being treated with a large range of pH: 2–13. In the comparison, pH stability of HDT was higher than that of the methanol extract of *Euonymus laxiflorus* Champ possessing relative activity of 48–55% at pH 4–8 [7], comparable to that of fermented nutrient broth (78–98%) [14], and lower than that of fermented squid pens (89–150%) [10]. These results suggest that HDT may be a good candidate for aGIs due to its potent activity and high pH stability.

### 2.3. Isolation and Identification of Active Compounds from MeOH Extract of HDT

**Figure 3:**
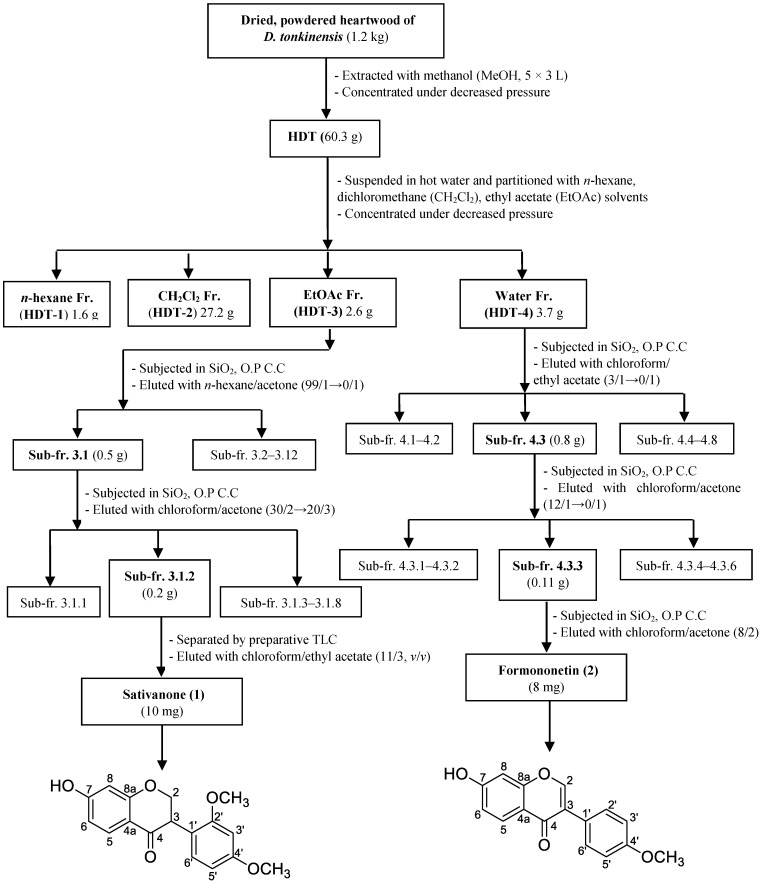
Flowchart for the isolation and identification of active inhibitors from the heartwood of *Dalbergia tonkinensis* (HDT); Fr. (fraction).

Various techniques, including solvent partition, silica open column chromatography (OPCC) and preparative TLC, coupled with a bioassay were used to isolate the active compounds from the heartwood of *Dalbergia tonkinensis*. The general process of these experiments is illustrated in Figure 3. 

#### 2.3.1. Primary Partitioned Separation of MeOH Extract of HDT

The MeOH extract of HDT was successively partitioned with *n*-hexane, dichloromethane, ethyl acetate and water to obtain 4 fractions. The yield and activity of HDT and its fractions are recorded in Table 2. HDT-3 partitioned by ethyl acetate demonstrated the strongest activity due to its greatest inhibition (%) and lowest EC_50_ value of 95% and 0.069 mg/mL, respectively. Thus, this fraction was chosen for the isolation of active compounds. The water fraction (HDT-4) showed acceptable activity. Since it had the good profile of TLC separation, it was also considered for subsequent experiments of purification.

#### 2.3.2. Sub-fractionation of HDT-3 and HDT-4, and Identification of Active Compounds

**Figure 4:**
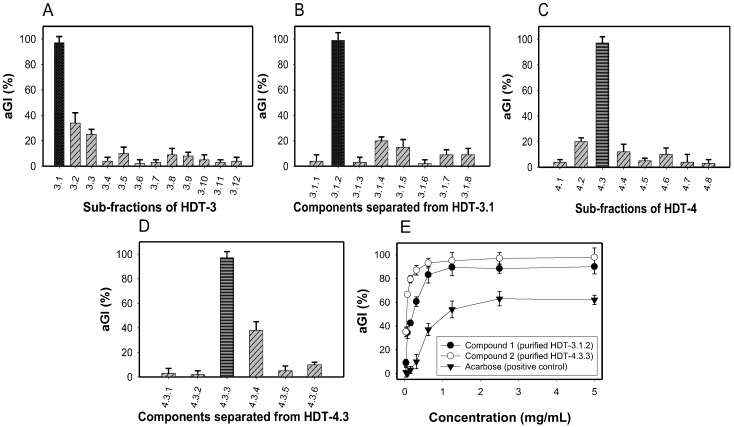
Inhibitory activity (%) of sub-fractions of HDT-3 (**A**) and HDT-4 (**C**) after separation by silica opened column (first time). aGI (%) of components of HDT-31 (**B**) and HDT-4.3 (**D**) after further separation by silica open column (second time). aGI (%) of isolated compounds and acarbose (**E**). Results are means ± SD of multi tests (*n* = 3).

The most active fraction (HDT-3) was separated by a silica open column to obtain 12 sub-fractions. The activity of these sub-fractions was tested and expressed as %. As shown in Figure 4A, HDT-3.1 showed the best activity (98%) while the others demonstrated weak inhibition (2–36%) at the same tested concentration of 0.1 mg/mL. Further separation of HDT-3.1 was applied to the most active sub-fraction (HDT-3.1.2), showing inhibition of 99% (Figure 4B).

HDT-4 fraction was also separated by a silica open column: 8 sub-fractions were obtained, their activity detected and then recorded in Figure 4C. The sub-fraction (HDT-4.3) exhibited good activity and as such was further separated via the same column. As shown in Figure 4D, the sub-fraction (HDT-4.3.3) possessed highest activity (98.5%) among 6 sub-fractions of HDT-4.3. 

For the two potent sub-fractions: HDT-3.1.2 and HDT-4.3.3, isolation of the active compounds by TLC was conducted. This final step of purification yielded 2 compounds: compound **1** (purified from HDT-3.1.2) and compound **2** (purified from HDT-4.3.3). These 2 active compounds were identified as sativanone (**1**) [31,32] and formononetin (**2**) [33,34], based on the analysis of NMR data and the comparison with reported compounds. Notably, sativanone (**1**) and formononetin (**2**) demonstrated much stronger activity than that of the positive control with the maximum activity and EC_50_ values of 90%, 0.23 mg/mL, 98 %, 0.059 mg/mL, and 62%, 1.321 mg/mL, respectively (Figure 4E).

Sativanone was reported to be distributed from the *Restharrow* root [35] and the heartwood of *Dalbergia odorifera* and showed several bioactivities including antibacterial activity against *Ralstonia solanacearum* [30] and anti-senescent and antioxidant effects [36]. This compound was also reported to possibly possess yeast α-glucosidase inhibition by using ultra-filtration liquid chromatography/mass spectrometry (UF-LC/MS) [37]. In this study, we strongly reconfirmed sativanone as a potent inhibitor by isolating sativanone and then testing its inhibition against yeast α-glucosidase. 

Formononetinis is a soy isoflavonoid that was isolated from several medicinal plants, including *Astragalus mongholicus* (Bunge), *Trifolium pretense* L. (red clover), *Butea monosperma* [33], *Euchresta formosana* [34], root and heartwood of *Dalbergia odorifera* [25]. Formononetin has also been reported to demonstrate some beneficial activities of yeast α-glucosidase inhibition, as well as antibacterial, antioxidant, anti-inflammatory, and cytotoxicity activities [25], to exhibit estrogenic properties and to promote angiogenesis [38].

The result highlighted that these efficient antidiabetic compounds were newly isolated from *Dalbergia tonkinensis* species and pre-confirmed *Dalbergia tonkinensis’* value to be developed as a health food due to its containing several bioactive compounds possessing vast beneficial bioactivities. In addition, the result of this study also enriches the current poor novel biological activities and chemical constituents of *Dalbergia tonkinensis* species.

### 2.4. Specific Inhibitory Activity of the Purified Compounds

Specific inhibition of the purified compounds was detected to characterize them as active antidiabetic drugs; a total of 4 enzymatic sources, including α-glucosidases from rat, yeast, bacterium, and rice were conducted to test the inhibition. The activity was expressed as EC_50_ value and presented in Table 3. The result indicated that sativanone showed efficient inhibition against bacterial α-glucosidase, and good inhibition against α-glucosidases from yeast and rat but had a weak effect on rice α-glucosidase. Formononetin also showed weak inhibition against rice α-glucosidase, good inhibition against rat α-glucosidase, and a very efficient effect on α-glucosidases from yeast and bacterium. Acarbose has the same ability of inhibition against rat and bacterial α-glucosidases as sativanone and formononetin, but very weak inhibition against yeast α-glucosidase, while showing an efficient effect on rice α-glucosidase. 

Yeast α-glucosidase has been used as the target enzyme in the screening of in vitro antidiabetic effect in many reports. However, α-glucosidase from rat was suggested as the better enzymatic source for evaluation of potent inhibitors since this enzyme is closer to that of human [14]. In this study, both sativanone and formononetin demonstrated much stronger inhibition against yeast α-glucosidase than did acarbose, and showed their effects on rat α-glucosidase comparable to that of acarbose. Thus, these two isolated compounds may be potential candidates for aGIs. In particular, the inhibitory activity of sativanone and formononetin against rat α-glucosidase was newly investigated in the current study, based on our current references review. Therefore, they were determined as new mammalian aGIs. 

### 2.5. The Rat α-Glucosidase Inhibitory Activity of Crude Extracts, Fractions, Sub-Fractions and Isolated Compounds from Dalbergia Tonkinensis

All the parts used extracts (the heartwood, trunk bark, and leaves) of *Dalbergia tonkinensis*, the fractions, sub-fractions, and purified compounds separated from HDT, as well as acarbose, were tested their inhibition against α-glucosidase from rat. The results were expressed as EC_50_ and max inhibition then summarized in Table 4. 

Among the 3 parts used extracts, HDT also demonstrated strongest inhibitory activity due to its smallest EC_50_ and greatest inhibition values of 1.72 µg/mL, and 61%, respectively. The activity was gradually increased via steps of partial purification. The 2 purified compounds showed their much stronger activity than that of the crude sample (HDT) and its fractions (HDT-3, HDT-4), and sub-fractions (HDT-3.1, HDT-3.1.2, HDT-4.3, and HDT-4.3.3). Based on Duncan’s multiple range test, these are no significant difference among the EC_50_ values of 2 isolated inhibitors (sativanone and formononetin) and that of acarbose as their EC_50_ values were ranged at ***fg***, ***fg***, and ***g*** level, respectively. In additional, they showed their same potency max inhibition (91–94%), and all their max inhibition values were ranked at the same level (***a***). Therefore, sativanone and formononetin were characterized as potent rat aGIs such as acarbose, a commercial antidiabetic drug. 

## 3. Materials and Methods

### 3.1. Chemicals and Reagents

Rice α-glucosidase (Type 4) was purchased from Sigma Aldrich, St. Louis City, MO, USA. Saccharomyces cerevisiae (yeast), B. stearothermophilus α-glucosidases and acarbose were obtained from Sigma Chemical Co., St. Louis City, MO, USA. Rat α-glucosidase was provided by Sigma Aldrich, Singapore. p-nitrophenyl glucopyranoside (pNPG) was purchased from Sigma Aldrich, 3050 Spruce Street, St. Louis, MO, USA. ^1^H-NMR (500 MHz) and ^13^C-NMR (125 MHz) were measured on a Bruker Avance 500 MHz spectrometer. Column chromatography was carried out on silica gel (Si 60 F_254_, 40–63 mesh, Merck, St. Louis, MO, USA). All solvents were redistilled before use. Pre-coated TLC plates (Si 60 F_254_) were used for analytical purposes. Compounds were visualized under UV radiation (254, 365 nm) and by spraying plates with 10% H_2_SO_4_, followed by heating with a heat gun. 

### 3.2. Plant Materials

Trunk bark, leaves and heartwood of *Dalbergia tonkinensis* (over ten years old) was collected in Buon Ma Thuot City, Daklak Province, Vietnam in 2016. The plant was identified by botanist Dr. Nguyen Quoc Binh, Vietnam National Museum of Nature, VAST, Hanoi, Vietnam, using the Checklist of plant species of Vietnam [39]. A voucher specimen (C-612) was deposited in the Department of Bioactive Products, Institute of Natural Products Chemistry, VAST, Hanoi, Vietnam.

### 3.3. Purification Procedures and Identification of Major α-Glucosidase Inhibitors

Dried powdered heartwood (1.2 kg) of Dalbergia tonkinensis was extracted at 60 °C with methanol (5 × 3.0 L) under reflux, filtered, and then concentrated under decreased pressure yielding a black crude methanol residue (60.3 g). The suspension of the methanol residue in hot water was successively partitioned with n-hexane, dichloromethane, and ethyl acetate to obtain n-hexane (1.6 g, HDT-1), dichloromethane (27.2 g, HDT-2), ethyl acetate (11.1 g, HDT-3) and water (12.0 g, HDT-4) fractions, respectively.

The fraction HDT-3 (2.6 g) was chromatographed on a normal silica gel (40–63 mesh) chromatography column (CC) using a gradient of n-hexane and acetone (99/1 to 0/1, *v*/*v*) as eluent to afford 12 sub-fractions (3.1-3.12). The sub-fraction 3.1 (500 mg) was rechromatographed on a normal silica gel CC using a gradient of chloroform-acetone (30/2 to 20/3, *v*/*v*) as eluent to produce 8 sub-fractions (3.1.1–3.1.8). The sub-fraction 3.1.2 (200 mg) was further separated by preparative TLC and eluting with chloroform-ethyl acetate (11/3, *v*/*v*) to obtain compound **1** (10.0 mg). 

The fraction HDT-4 (3.7 g) was chromatographed on a normal silica gel (40-63 mesh) chromatography column (CC) using a gradient of chloroform-ethyl acetate (3/1 to 0/1, *v*/*v*) as eluent to afford 8 sub-fractions (4.1–4.8). The sub-fraction 4.3 (800 mg) was rechromatographed on a normal silica gel CC using a gradient of chloroform-acetone (12/1 to 0/1, *v*/*v*) as eluent to produce 6 sub-fractions (4.3.1–4.3.6). Compound **2** (8.0 mg) was obtained from the sub-fraction 4.3.3 (110 mg) by a normal silica gel CC, eluting with chloroform-acetone (8/2). 

Sativanone (**1**) was obtained as white amorphous powder; ^1^H-NMR (500 MHz, Acetone-*d*_6_) *δ*_H_: 9.40 (1H, br s, 7-OH), 7.77 (1H, d, *J* = 9.0 Hz, H-5), 7.01 (1H, d, *J* = 8.5 Hz, H-6′), 6.59 (1H, d, *J* = 2.5 Hz, H-8), 6.58 (1H, dd, *J* = 9.0, 2.5 Hz, H-6), 6.48 (1H, dd, *J* = 8.5, 2.5 Hz, H-5′), 6.40 (1H, d, *J* = 2.5 Hz, H-3′), 4.56 (1H, t, *J* = 11.0 Hz, H_a_-2), 4.45 (1H, dd, *J* = 11.0, 5.5 Hz, H_b_-2), 4.18 (1H, dd, *J* =11.0, 5.5 Hz, H-3), 3.79 (3H, s, 2′-OCH_3_), 3.78 (3H, s, 4′-OCH_3_). ^13^C-NMR (125 MHz, Acetone-*d*_6_) *δ*_C_: 190.7 (s, C-4), 164.5 (s, C-7), 164.3 (s, C-8a), 161.1 (s, C-4′), 159.1 (s, C-2′), 131.2 (d, C-6′), 129.7 (d, C-5), 117.0 (s, C-1′), 110.8 (d, C-6), 105.3 (d, C-5′), 103.1 (d, C-3′), 99.2 (d, C-8), 71.4 (t, C-2), 55.6 (q, 4′-OCH_3_), 55.2 (q, 2′-OCH_3_), 47.6 (d, C-3).

Formononetin (**2**) was obtained as white amorphous powder; ^1^H-NMR (500 MHz, Methanol-*d*_4_) *δ*_H_: 8.17 (1H, s, H-2), 8.08 (1H, d, *J* = 8.5 Hz, H-5), 7.49 (2H, d, *J* = 8.5 Hz, H-2′, H-6′), 7.01 (2H, d, *J* = 8.5 Hz, H-3′, H-5′), 6.97 (1H, dd, *J* = 8.5, 2.0 Hz, H-6), 6.88 (1H, d, *J* = 2.0 Hz, H-8), 3.85 (3H, s, 4′-OCH_3_); ^13^C-NMR (125 MHz, Methanol-*d*_4_): 178.1 (s, C-4), 164.9 (s, C-7), 161.1 (s, C- C-4′), 159.8 (s, C-8a), 154.8 (d, C-2), 131.4 (d, C-2′, C-6′), 128.5 (d, C-5), 125.7 (s, C-3), 125.6 (s, C-1′), 118.1 (s, C-4a), 116.6 (d, C-6), 114.9 (d, C-3′, C-5′), 103.3 (d, C-8), 55.7 (q, 4′-OCH_3_).

### 3.4. Enzymatic Inhibitory Assays

The inhibitory activity against α-glucosidases was done according to the assay described by Nguyen, et al. 2018 [15]. The mixture of 50 µL of the sample, 50 µL of the α-glucosidase solution and 100 µL potassium phosphate buffer was pre-incubated at 37 °C for 20 min for the combination of inhibitors with enzymes. 50 µL of substrate (*p*NPG) was added into the mixture to start the reaction. This reaction step was maintained at 37 °C for 30 min for the detection of inhibition against α-glucosidases from rice, yeast, and bacteria, and for 60 min when rat α-glucosidases was used instead. Thereafter, the mixture solution’s optical density was detected at 405 nm (the wavelength). The following formula was used to calculate the α-glucosidase inhibitory activity:

**aGI** (%) = (**A** − **B**)/**A** × 100%, (1)

where **aGI** is α-glucosidase inhibitory activity, **A** and **B** are the optical density of the reaction mixture without sample (inhibitor) and the reaction mixture presenting sample, respectively, at the wavelength 405nm. The concentration of inhibitor that inhibits 50% of activity of α-glucosidase under the assay conditions was defined as the EC_50_ value. Potassium phosphate buffer (0.1 mol/L, pH 7) was used for the preparation of the enzymes, substrate, and sample solutions. The rat α-glucosidase solution was prepared according to the previous report [14]. Bacterial, yeast and rice glucosidases were tested at the concentrations of 1.0, 0.25, and 0.1 U/mL, respectively.

## 4. Conclusions

The methanol extracts of all the parts of *Dalbergia tonkinensis* used, including heartwood, trunk bark, and leaves, were found to be potential sources of α-glucosidase inhibitors (aGIs), showing more efficient inhibitory activity than that of acarbose. The 2 active inhibitors were successfully isolated and identified from the heartwood of *Dalbergia tonkinensis*. Notably, these active compounds, sativanone and formononetin demonstrated much stronger activity than that of acarbose. The specific inhibitory activity tests showed that sativanone and formononetin also have good effects on mammalian α-glucosidase; this property of these inhibitors was newly investigated in this study. The results highlight that *Dalbergia tonkinensis* is a valuable source of aGIs and could be developed as a health food due to it containing several compounds possessing various beneficial bioactivities. These results also enrich the current poor understanding of the novel biological activities and constituents of *Dalbergia tonkinensis* species. However, the mechanism of enzyme inhibition of the active compounds described here is still unknown, as such it will be important to investigate in further studies.

## Authors Contributions

Conceived the study: S.L.W., N.M.C., V.B.N., N.T.N. Designed the study: V.B.N., S.L.W., N.M.C. Performed the experiments: V.B.N., N.T.N., T.H.N. Contributed reagents/materials/analysis tools: S.L.W., N.M.C., V.B.N., P.D.N.N. Analyzed data: V.B.N., S.L.W., N.M.C., T.H.N., N.T.N., P.D.N.N, D.H.N. Wrote the paper: V.B.N., S.L.W., N.M.C.

## Figures and Tables

**Table 1:** α-glucosidase inhibition of some potential extract of medicinal plants collected in Dak Lak.

No.	Scientific Name of Medicinal Plants	Part Used	EC_50_ (mg/mL)	References
*1*	*Dalbergia tonkinensis*	Heartwood	0.17 ± 0.013	This study
	*Acabose* (*positive control*)		1.21 ± 0.103	
*2*	*Terminalia alata*	Trunk bark	≥4	Nguyen et al., 2016 [8]
*3*	*Terminalia bellirica*	Trunk bark	0.41 ± 0.03	Nguyen et al., 2016 [8]
*4*	*Terminalia corticosa*	Trunk bark	1.42 ± 0.02	Nguyen et al., 2016 [8]
*5*	*Euonymus laxiflorus Champ.*	Trunk bark	0.360 ± 0.03	Nguyen et al., 2017 [9]
*6*	*Euonymus laxiflorus Champ.*	Leaves	0.67	Nguyen et al., 2017 [7]
*7*	*Cinnamomum cassia J. S. Presl.*	Trunk bark	1.08	Nguyen et al., 2017 [7]
*8*	*Terminalia bellirica*	Leaves	0.66	Nguyen et al., 2017 [7]
*9*	*Psidium littorale Raddi*	Leaves	0.25 ± 0.01	Nguyen et al., 2018 [22]

All the samples were extracted with methanol as the method described in the material and method section; their inhibition against α-glucosidase was tested and expressed as EC_50_ value.

**Table 2:** α-glucosidase inhibition of HDT and its fractions after partition.

Samples		Yield (g)	α-Glucosidase Inhibition	
			EC_50_ (mg/mL)	Inhibition (%) *
HDT	*(crude MeOH extract)*	60.3	0.172 ± 0.011	98 ± 3.2
HDT-1	*(Hexane Fr.)*	1.6	1.712 ± 0.210	73 ± 4.1
HDT-2	*(Dichloromethane Fr.)*	27.2	0.124 ± 0.003	90 ± 2.5
HDT-3	*(Ethyl acetate Fr.)*	11.1	0.069 ± 0.001	95 ± 3.7
HDT-4	*(Water Fr.)*	12.0	0.513 ± 0.051	82 ± 2.3
Acarbose	*(positive control)*		1.357 ± 0.03	62 ± 1.8

*: the inhibition of acarbose and fractions were detected at their concentration range of 0.1–5 mg/mL; results are means ± SD of multi tests (*n* = 3).

**Table 3:** Specific inhibitory activity of Sativanone, Formononetin, and Acarbose.

No.	Enzyme Source	Inhibition Expressed as EC_50_ (mg/mL)		
		Sativanone	Formononetin	Acarbose
1	Yeast α-glucosidase	0.23 ± 0.012	0.06 ± 0.002	1.321 ± 0.048
2	Rat α-glucosidase	0.37 ± 0.022	0.23 ± 0.037	0.121 ± 0.001
3	Bacterial α-glucosidase	0.07 ± 0.001	0.03 ± 0.002	0.001 ± 0.000
4	Rice α-glucosidase	0.81 ± 0.023	0.98 ± 0.029	0.031 ± 0.005

All tests were performed in triplicate; results are means ± SD of multi tests (*n* = 3).

**Table 4:** Rat α-glucosidase inhibitory activity of crude extracts, fractions, sub-fractions, and isolated compounds from Dalbergia tonkinensis extract.

Components	Rat α-glucosidase Inhibitory Activity	
	EC_50_ (mg/mL)	Max Inhibition (%)
**Heartwood Extract (HDT)**	1.72 ± 0.116 *^b^*	61 ± 3.46 *^e^*
Trunk bark extract	2.91 ± 0.289 *^a^*	51 ± 4.62 *^f^*
Leaves extract	2.78 ± 0.173 *^a^*	54 ± 4.60 *^f^*
HDT-3	1.31 ± 0.057 *^c^*	68 ± 5.77 *^d^*
HDT-3.1	1.13 ± 0.058 *^cd^*	75 ± 5.20 *^c^*
HDT-3.1.2	0.92 ± 0.023 *^d^*	77 ± 5.18 *^c^*
**Sativanone**	0.357 ± 0.006 *^fg^*	91 ± 4.61 *^a^*
HDT-4	1.43 ± 0.115 *^bc^*	67 ± 2.89 *^d^*
HDT-4.3	0.87 ± 0.035 *^de^*	78 ± 4.61 *^c^*
HDT-4.3.3	0.55 ± 0.012 *^ef^*	84 ± 4.62 *^b^*
**Formononetin**	0.251 ± 0.006 *^fg^*	94 ± 5.11 *^a^*
***Acarbose***	*0.119 ± 0.005 ^g^*	*93 ± 2.5 ^a^*
Coefficient of variation	12.50026	1.853018

The samples and acarbose were tested at their concentration range of 0.05–3.2 mg/mL; results are means ± SD of multi tests (*n* = 3); the means of EC_50_ and max inhibition values with the different letter in the same column are significantly different in comparison based on Duncan’s multiple range test (alpha = 0.01) using SAS version 9.4, Statistical Analysis Software analysis.
